# P-574. Extrapolating Expected Efficacy, Safety, and Pharmacokinetics (PK) of Dolutegravir/Lamivudine (DTG/3TC) in Pregnant People Living with HIV-1 from Pregnancy Data with Combination DTG and/or 3TC Antiretroviral Therapies

**DOI:** 10.1093/ofid/ofae631.772

**Published:** 2025-01-29

**Authors:** William R Short, Parul Patel, Gustavo Verdier, Ana Puga, Andrew Weber, Leigh Ragone, Eva Fernvik, Vani Vannappagari, V Paul DiMondi, Clifford B Jones

**Affiliations:** University of Pennsylvania, Philadelphia, Pennsylvania; ViiV Healthcare, Durham, North Carolina; ViiV Healthcare, Montréal, QC, Canada, Pointe-Claire, Quebec, Canada; ViiV Healthcare, Durham, North Carolina; ViiV Healthcare, Durham, North Carolina; ViiV Healthcare, Durham, North Carolina; ViiV Healthcare, Durham, North Carolina; ViiV Healthcare, Durham, North Carolina; ViiV Healthcare, Durham, North Carolina; ViiV Healthcare, Brentford, UK, London, England, United Kingdom

## Abstract

**Background:**

Minimizing fetal exposure to unnecessary drugs is a guiding principle during pregnancy. DTG/3TC has demonstrated efficacy, a high barrier to resistance, and a favorable safety profile. Data on DTG/3TC use in pregnant people living with HIV-1 are limited; however, safety and PK data for the individual components are extensive from DTG- and 3TC-containing 3-drug regimens (3DRs) as preferred regimens in pregnancy. Randomized trial data and PK from pregnant participants receiving DTG 3DRs or 2-drug regimens were used to extrapolate anticipated DTG/3TC efficacy, safety, and PK in pregnancy.

**Methods:**

A literature review of articles, conferences, Antiretroviral Pregnancy Registry (APR) Interim Summary, and other non-interventional studies including DTG and/or 3TC in PubMed (Jan 1998-Nov 2023) using the search terms dolutegravir, lamivudine, and pregnancy was conducted. Efficacy, safety, PK, vertical transmission, and birth outcomes data were extracted. Clinical utility of DTG/3TC in pregnancy was evaluated in context of current perinatal treatment guidelines.

**Results:**

Two studies evaluated DTG/3TC efficacy and safety in pregnancy: 30/31 (97%) participants maintained virologic suppression (< 50 c/mL); no vertical transmission occurred. Among 194 studies of DTG and 492 of 3TC, 3 randomized trials of DTG 3DR efficacy and safety in pregnancy were found. While DTG PK in pregnancy was generally lower and 3TC PK was slightly lower than postpartum, both were well above thresholds associated with efficacy in non-pregnant adults. Overall birth defect prevalence rate with DTG exposure at conception was 0.95% (16/1683, Tsepamo) and 0.88% (43/4902, Eswatini). The APR reported overall birth defect prevalence of 3.3% (32/957) with first-trimester and 5.3% (29/549) with second/third-trimester DTG exposure, which did not significantly differ from the population-expected rate (Metropolitan Atlanta Congenital Defects Program, 2.7%; Texas Birth Defects Registry, 4.2%).

**Conclusion:**

DTG/3TC is anticipated to be similarly effective and tolerated for parental health and vertical transmission prevention with fetal exposure to fewer drugs. Pregnancy is not anticipated to significantly impact DTG/3TC exposure. DTG and 3TC are well tolerated with a well-established safety profile in pregnancy.

**Disclosures:**

**William R. Short, MD**, Gilead: Grant/Research Support|Janssen: Honoraria|ViiV Healthcare: Advisor/Consultant|ViiV Healthcare: Honoraria **Parul Patel, MSW**, GSK: Stocks/Bonds (Public Company)|ViiV Healthcare: Employee **Gustavo Verdier, BSc, BPharm, MBA**, ViiV Healthcare: Employee **Ana Puga, MD, FAAP, AAHIVS**, GSK: Stocks/Bonds (Public Company)|ViiV Healthcare: Employee **Andrew Weber, MS**, GSK: Stocks/Bonds (Public Company)|ViiV Healthcare: Employee **Leigh Ragone, MS**, GlaxoSmithKline: Stocks/Bonds (Public Company)|ViiV Healthcare: Full time employee **Eva Fernvik, PhD**, GSK: Stocks/Bonds (Public Company)|ViiV Healthcare: Employee **Vani Vannappagari, MBBS, MPH, PhD**, GSK: Stocks/Bonds (Public Company)|ViiV Healthcare: Full time Employee|ViiV Healthcare: Stocks/Bonds (Public Company) **V. Paul DiMondi, PharmD**, GSK: Stocks/Bonds (Public Company)|ViiV Healthcare: Employee **Clifford B. Jones, BSc MSc MB ChB**, GSK: Stocks/Bonds (Public Company)|ViiV Healthcare: Employee

